# A real‐world comparison between neoadjuvant chemoimmunotherapy and chemotherapy alone for resectable non‐small cell lung cancer

**DOI:** 10.1002/cam4.4889

**Published:** 2022-05-27

**Authors:** Baihua Zhang, Haifan Xiao, Xingxiang Pu, Chunhua Zhou, Desong Yang, Xu Li, Wenxiang Wang, Qin Xiao

**Affiliations:** ^1^ The Second Department of Thoracic Surgery, Hunan Clinical Medical Research Center of Accurate Diagnosis and Treatment for Esophageal Carcinoma, Hunan Cancer Hospital and The Affiliated Cancer Hospital of Xiangya School of Medicine Central South University Changsha People's Republic of China; ^2^ Cancer Prevention Office, Hunan Cancer Hospital and The Affiliated Cancer Hospital of Xiangya School of Medicine Central South University Changsha People's Republic of China; ^3^ The Second Department of Thoracic Medical Oncology, Hunan Cancer Hospital and The Affiliated Cancer Hospital of Xiangya School of Medicine Central South University Changsha People's Republic of China; ^4^ Department of Medical Oncology, Lung Cancer and Gastrointestinal Unit, Hunan Cancer Hospital and The Affiliated Cancer Hospital of Xiangya School of Medicine Central South University Changsha People's Republic of China; ^5^ Key Laboratory of Translational Radiation Oncology, Hunan Province; The First Department of Thoracic Radiation Oncology, Hunan Cancer Hospital, The Affiliated Cancer Hospital of Xiangya School of Medicine Central South University Changsha People's Republic of China

**Keywords:** chemoimmunotherapy, neoadjuvant therapy, non‐small cell lung cancer, sleeve lobectomy, surgery

## Abstract

**Background:**

The impact of neoadjuvant chemoimmunotherapy on pulmonary resection and related outcomes had been poorly reported in previous studies. The present study aims to clarify the efficacy and safety of neoadjuvant chemoimmunotherapy, and intraoperative difficulty in the following surgery, in comparison with chemotherapy alone in non‐small cell lung cancer (NSCLC).

**Methods:**

Patients with newly diagnosed clinical stages IB–IIIB(T3‐4N2) NSCLC, received neoadjuvant chemotherapy + PD‐1 inhibitors (PD‐1 + Chemo group) or chemotherapy alone (Chemo group) followed by surgery between December 2018 and December 2020 were included. The clinicopathological characteristics were retrospectively reviewed and analyzed.

**Results:**

There were 69 NSCLC patients in the PD‐1 + Chemo group and 121 in the Chemo group. The major pathological response (MPR) rate in the PD‐1 + Chemo group was 49.3%, higher than that of 19.0% in the Chemo group (*p* < 0.001). The 2‐year disease‐free survival (DFS) rate was 79.3% and 60.2%, respectively, in the two groups (*p* = 0.048). Multivariate analysis identified surgical radicality (hazard ratio (HR), 2.954, 95% confidence interval (CI), 1.527–5.714, *p* = 0.001), and pathological response (MPR(CR) vs. SD(PD), HR, 0.248, 95% CI, 0.107–0.572, *p* = 0.001) to be independent prognostic factors for DFS. Lobectomy was performed in 73.9% and 66.1% of patients, respectively, and bronchial sleeve resection/bronchoplasty rate was also comparable (43.4% vs. 40.5%, *p* = 0.688). More patients in the PD‐1 + Chemo group received vascular sleeve resection/angioplasty (15.9% vs. 6.6%, *p* = 0.039) and pericardial resection (10.1% vs. 2.5%, *p* = 0.038). After propensity score matching analysis, pericardial resection rate was still slightly higher in the PD‐1 + Chemo group (9.4% vs. 1.6%, *p* = 0.05). Perioperative morbidities within 30 days and mortality in 90 days were comparable between groups (*p* > 0.05).

**Conclusions:**

Neoadjuvant chemoimmunotherapy for NSCLC is safe and feasible, with higher MPR rates, as well as favorable DFS than chemotherapy alone. Surgical complexity might be increased in certain patients, with comparable perioperative morbidity and mortality.

## Graphical abstract

This real‐world study confirmed that neoadjuvant chemoimmunotherapy is safe and feasible, with higher MPR and pCR rates, as well as favorable DFS compared with chemotherapy alone. Surgical complexity might be increased in certain patients, with comparable perioperative morbidity and mortality. This large‐scale real‐word study has provided a solid evidence for the use of neoadjuvant chemoimmunotherapy for NSCLC in the future. Our results may help improve the perioperative management and surgical techniques in pulmonary resections following neoadjuvant chemoimmunotherapy.

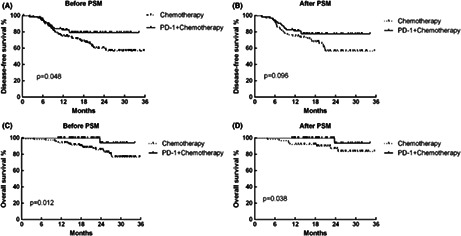

## 1 INTRODUCTION

Lung cancer remains the leading cause of cancer‐related deaths worldwide, and 85% of newly diagnosed cases are of non‐small cell lung cancer (NSCLC).^1^ In early‐stage lung cancer, the primary purpose of treatment is to cure. Surgery remains a cornerstone of curative treatment for operable NSCLC, with a 5‐year overall survival (OS) rate between 92% in stage IA and 26% in stage IIIB patients.^2^ However, almost 30% to 55% of patients suffer from recurrence, especially distant metastasis, within 5 years, even after complete resection.^3^ Neoadjuvant or adjuvant chemotherapy might achieve a better prognosis, but with only a 5% improvement of the 5‐year OS.^4^, ^5^ Therefore, multidisciplinary treatment modalities including surgery in combination with the associated systemic therapies might further improve the likelihood of a cure.

Recently, neoadjuvant immune checkpoint inhibitors (ICI) including programmed cell death‐ligand 1 (PD‐L1) and programmed cell death protein‐1 (PD‐1) inhibitors have proven effective for locally advanced NSCLC. A systematic review^6^ that included 19 phase I–III clinical studies indicated that neoadjuvant immunotherapy achieved better pathological responses, especially in combination with chemotherapy. After neoadjuvant therapy using mono ICI, dual therapy‐ICI, chemoradiation‐ICI, radiotherapy‐ICI, or chemo‐ICI, the pathological complete response (pCR) rates were 7% to 16%, 33% to 38%, 27%, 27%, and 9% to 63%, respectively.^6^ The Checkmate 816 trial has been the only phase III prospective trial that has published results so far, showing a 21.6% increase in the pCR rate of patients treated with neoadjuvant Nivolumab + platinum doublets compared with that of patients treated with chemotherapy alone.^7^ As a result, the use of PD‐1/PD‐L1 inhibitors in the neoadjuvant setting has revolutionized the treatment of early‐stage NSCLC. Currently, numerous prospective phase II/III clinical trials are underway to further investigate the efficacy of neoadjuvant chemoimmunotherapy compared with that of chemotherapy alone in resectable NSCLC.^8^, ^9^

The main concerns for surgery following neoadjuvant immunotherapy are related to challenges in the perioperative management and surgical techniques it poses. The prospective surgical data from the Checkmate 816 study^7^ showed that the feasibility, duration of surgery, definitive surgery rates, and surgical complications after neoadjuvant Nivolumab + chemotherapy were not negatively influenced. However, these well‐designed clinical trials are far from enough to reflect the complicated situations in real clinical practice. There are still worries about the severe adverse events (AEs) associated with ICIs, which might compromise definitive surgery, the risk of disease progression before surgery, the potential for increased intraoperative difficulty, and greater perioperative complications, especially in potentially resectable NSCLC cases.^10^ Therefore, more large‐scale phase III clinical trials and real‐world studies are required to evaluate the impact of preoperative chemoimmunotherapy on surgery.

In this study, we conducted a real‐world retrospective analysis of 190 NSCLC patients who received neoadjuvant chemoimmunotherapy or chemotherapy alone. To balance the heterogeneity between groups, propensity score matching (PSM) was used in the analysis. The primary aim was to discuss the efficacy of neoadjuvant chemoimmunotherapy and surgical difficulty in following pulmonary resections in comparison with chemotherapy alone. We present the following article according to the (STROBE) guideline checklist.

## 2 PATIENTS AND METHODS

### 2.1 Patients' selection

The inclusion criteria for this study were as follows: patients who were newly diagnosed with clinical stage IB–IIIB (T3‐4N2) (American Joint Committee on Cancer, 8th edition) NSCLC and received neoadjuvant chemotherapy (±PD‐1 inhibitors) (one cycle at least) followed by surgery between December 2018 and December 2020 at Hunan Cancer hospital. All patients had an Eastern Cooperative Oncology Group (ECOG) performance status (PS) score of 0 or 1. Patients were excluded based on the following criteria: (1) pathological non‐NSCLC components included; (2) patients with distant metastasis before neoadjuvant treatment; (3) patients who received other therapies prior to surgery except neoadjuvant chemotherapy (±PD‐1 inhibitors); (4) patients with a history of previous cancers or other concurrent malignant diseases; (5) patients who failed to undergo surgery for any reason. In total, there were 190 patients included in this study, including 69 patients who received neoadjuvant PD‐1 inhibitors plus chemotherapy, and 121 patients who received neoadjuvant chemotherapy alone. Detailed demographic and clinicopathological characteristics were retrospectively retrieved from medical records and analyzed.

The study was conducted in accordance with the Declaration of Helsinki (as revised in 2013). As a retrospective real‐world observational study, the Ethics Committee of Hunan Cancer Hospital approved the study, and written informed consent from patients was waived (No. 2022‐18).

### 2.2 Treatment methods

On the basis of the neoadjuvant therapeutic modality, 69 patients who were treated with neoadjuvant PD‐1 inhibitors plus chemotherapy were divided into the PD‐1 + Chemo group, and the other 121 patients who were treated with chemotherapy alone were divided into the Chemo group. All patients received conventional platinum‐based (cisplatin or carboplatin) doublets chemotherapy (21 days per cycle). One of the following PD‐1 inhibitors was given intravenously in the PD‐1 + Chemo group according to the international consensus: Pembrolizumab (18 cases, 26.1%), Toripalimab (16 cases, 23.2%), Sintilizumab (13 cases, 18.8%), Nivolizumab (9 cases, 13.0%), Camrelizumab (8 cases, 11.6%), and Tislelizumab (5 cases, 7.2%).

After discussion by a multidisciplinary team including thoracic surgeons, curative‐intent resection was performed after recovery from neoadjuvant treatment. Surgery performed beyond 42 days after the final neoadjuvant therapy was defined as delayed surgical resection in the present study. Some patients received pulmonary resection with systemic lymphadenectomy via minimally invasive surgery (video‐assisted thoracic surgery [VATS]) or traditional open thoracotomy. Definitive resection was defined according to the standards reported by Rami‐Porta et al.^11^

Postoperative treatment was administered according to the recovery condition of each patient, treatment‐related adverse events (TRAEs), and pathological responses after the discussion of the multidisciplinary team. In the PD‐1 + Chemo group, adjuvant PD‐1 inhibitors might be recommended until month 12 after four cycles of chemoimmunotherapy (including neoadjuvant and adjuvant chemoimmunotherapy in total).

### 2.3 Tumor evaluation and follow‐up

Radiographic evaluations were performed at the initial diagnosis, before surgery, every 3 months during the first 2 years after surgery, and then every 6 months thereafter, including ^18^F‐FDG positron emission tomography–computed tomography (PET‐CT) or/and chest and abdominal contrast tomography (CT), brain magnetic resonance imaging (MRI), cervical and heart ultrasound, and ^99^Tcm‐MDP bone scan. Radiographic tumor responses were assessed before surgery according to the Response Evaluation Criteria in Solid Tumors (RECIST, version 1.1). Whenever recurrence was suspected, ^18^F‐FDG PET‐CT, contrast‐enhanced CT, or MRI were performed to confirm the recurrence.

Pathological treatment responses were graded according to the histopathologic response criteria reported by Pataer et al.^12^ and Cottrell et al.^13^ Major pathological response (MPR) was defined as no more than 10% viable residual tumor cells identified in the resected specimen; pCR was defined as no viable tumor cells identified in the specimen. TRAEs were graded in accordance with the National Cancer Institute Common Terminology Criteria for Adverse Events version 5.0.

### 2.4 Statistical analysis

The primary endpoint was the MPR rate, and the secondary endpoints were the rate of delayed surgery, radical resection (R0), perioperative morbidity, and 2‐year disease‐free survival (DFS). DFS was calculated from the date of surgery to the date of confirmed disease progression or death, analyzed by the Kaplan–Meier method, and compared with the log‐rank test.

Heterogeneity in baseline clinical characteristics (age, gender, smoking habits, weight loss before treatment, tumor location, histology, cT stage, cN stage, and cTNM) before treatment between the PD‐1 + Chemo and Chemo groups was balanced by PSM using the nearest‐neighbor method with a ratio of 1:1, without replacement, and with a 0.02‐caliper width. Standardized mean differences were used to assess the balance of covariate distributions between treatment groups before and after matching. Differences in demographic and clinicopathological characteristics between groups were assessed by the chi‐square (*χ* ^2^) test for categorical variables, the Wilcoxon rank sum test for ordinal continuous variables, or t‐test for continuous variables. Survival analyses were conducted using Kaplan–Meier curves and compared by the log‐rank test. Statistical analyses were performed using SPSS 23.0 (IBM Corp.). *p* < 0.05 (two‐sided) was considered statistically significant.

## 3 RESULTS

### 3.1 Baseline characteristics of the patient cohort

There were 69 NSCLC patients in the PD‐1 + Chemo group and 121 in the Chemo group. Baseline demographic and clinicopathological characteristics are summarized in Table 1. The percentages of patients aged ≥60 in each group were 46.4%, and 52.9%, respectively (*p* = 0.388). No significant gender difference was detected between the two groups, both in which men were predominant, 95.7% and 90.1%, respectively (*p* = 0.171). There were no significant differences between the two groups in smoking habits, weight loss before treatment, tumor location, cN stage, or cTNM stage before treatment. The percentage of patients with stage T1–2 disease in the PD‐1 + Chemo group was 40.6%, which was significantly lower than in the Chemo group (57.0%; *p* = 0.029). The principal histological type in both groups was squamous cell carcinoma (52 cases [75.4%] and 89 cases [73.6%], respectively; *p* = 0.784). Additionally, there were cases of non‐squamous cell carcinoma including adenocarcinoma (16 cases, 23.2%) and adenosquamous cell carcinoma (1 case, 1.4%) in the PD‐1 + Chemo group and 31 adenocarcinomas (25.6%) and one sarcomatoid carcinoma (0.8%) in the Chemo group.

**TABLE 1:** Baseline clinical characteristics of NSCLC patients treated with neoadjuvant therapy and PSM analysis

Characteristic	Before PSM				After PSM		
	PD‐1 + Chemo *N* = 69 (%)	Chemo *N* = 121 (%)	*p* value	SMD^a^	PD‐1 + Chemo *N* = 64 (%)	Chemo *N* = 64 (%)	SMD^a^
Age							
<60	37 (53.6)	57 (47.1)	0.388	0.131	33 (51.6)	30 (46.9)	0.093
≥60	32 (46.4)	64 (52.9)			31 (48.4)	34 (53.1)	
Gender							
Male	66 (95.7)	109 (90.1)	0.171	0.218	61 (95.3)	60 (93.8)	0.068
Female	3 (4.3)	12 (9.9)			3 (4.7)	4 (6.3)	
Smoking habits							
Non‐smoker	7 (10.1)	21 (17.4)	0.178	0.211	7 (10.9)	9 (14.1)	0.094
Present/ex‐smoker	62 (89.9)	100 (82.6)			57 (89.1)	55 (85.9)	
Weight loss before treatment							
Yes	15 (21.7)	32 (26.4)	0.470	0.184	14 (21.9)	22 (34.4)	<0.001
No	54 (78.3)	89 (73.6)			50 (78.1)	42 (65.6)	
Tumor location							
Peripheral	17 (24.6)	26 (21.5)	0.618	0.075	14 (21.9)	14 (21.9)	<0.001
Central	52 (75.4)	95 (78.5)			50 (78.1)	50 (78.1)	
Histology							
Squamous cell carcinoma	52 (75.4)	89 (73.6)	0.784	0.041	49 (76.6)	48 (75.0)	0.036
Non squamous cell carcinoma	17 (24.6)	32 (26.4)			15 (23.4)	16 (25.0)	
Tumor length (cm)	5.11±1.88	4.80±2.18	0.327	0.151	4.98±1.87	5.06±2.00	0.044
cT stage							
T1–2	28 (40.6)	69 (57.0)	0.029	0.334	28 (43.8)	25 (39.1)	0.095
T3–4	41 (59.4)	52 (43.0)			36 (56.3)	39 (60.9)	
cN stage							
N0	10 (14.5)	15 (12.4)	0.202	0.061	9 (14.1)	11 (17.2)	0.085
N1	27 (39.1)	34 (28.1)			24 (37.5)	21 (32.8)	
N2	32 (46.4)	72 (59.5)			31 (48.4)	32 (50.0)	
cTNM stage							
IB–II	17 (24.6)	31 (25.6)	0.597	0.109	16 (25.0)	16 (25.0)	0.022
IIIA	34 (49.3)	64 (52.9)			31 (48.4)	32 (50.0)	
IIIB (T3‐4N2)	18 (26.1)	26 (21.5)			17 (26.6)	16 (25.0)	

Abbreviations: Chemo, chemotherapy; cN, clinical N stage before treatment; cT, clinical T stage before treatment; cTNM, clinical TNM stage before treatment; NSCLC, non‐small cell lung cancer; PD‐1 + Chemo, PD‐1 inhibitor plus chemotherapy; PSM, propensity score matching.

^a^ Imbalance between treatment groups was defined as a SMD ≥0.1; balance between treatment groups was defined as a SMD <0.1.

In PSM analysis, the following nine baseline factors including age, gender, smoking habits, weight loss before treatment, tumor location, histology, cT stage, cN stage, and cTNM were well balanced between the two groups (Table 1).

### 3.2 Surgical treatment

All patients received one to five cycles of neoadjuvant therapy before surgery, and 14 patients (20.3%) and 27 patients (22.3%), respectively, received ≥3 cycles of neoadjuvant therapy in the two groups (Table 2, *p* = 0.744). The frequency of carboplatin treatment was 87.0% in the PD‐1 + Chemo group, which was significantly higher than in the Chemo group (65.3%; *p* = 0.001).

**TABLE 2:** Comparison of treatment modality and surgical outcomes in NSCLC population

Characteristics	Before PSM			After PSM		
	PD‐1 + Chemo *N* = 69 (%)	Chemo *N* = 121 (%)	*p*	PD‐1 + Chemo *N* = 64 (%)	Chemo *N* = 64 (%)	*p*
Neoadjuvant therapy cycles						
<3	55 (79.7)	94 (77.7)	0.744	50 (78.1)	50 (78.1)	1.000
≥3	14 (20.3)	27 (22.3)		14 (21.9)	14 (21.9)	
Platinum type						
Cisplatinum	9 (13.0)	42 (34.7)	0.001	8 (12.5)	20 (31.3)	0.010
Carboplatin	60 (87.0)	79 (65.3)		56 (87.5)	44 (68.8)	
TRAEs related to drugs						
Yes	41 (59.4)	69 (57.0)	0.748	25 (39.1)	27 (42.2)	0.719
No	28 (40.6)	52 (43.0)		39 (60.9)	37 (57.8)	
Interval between final neoadjuvant therapy and surgery, days						
≤42 days	59 (85.5)	107 (88.4)	0.560	55 (85.9)	55 (85.9)	1.000
>42 days	10 (14.5)	14 (11.6)		9 (14.1)	9 (14.1)	
Surgery approach						
VATS	27 (39.1)	38 (31.4)	0.187	24 (37.5)	16 (25.0)	0.237
Conversion	20 (29.0)	28 (23.1)		19 (29.7)	19 (29.7)	
Thoracotomy	22 (31.9)	55 (45.5)		21 (32.8)	29 (45.3)	
Extent of resection						
Lobectomy	51 (73.9)	80 (66.1)	0.471	47 (73.4)	45 (70.3)	0.524
Bilobectomy	10 (14.5)	29 (24.0)		9 (14.1)	13 (20.3)	
Pneumonectomy	7 (10.1)	11 (9.1)		7 (10.9)	5 (7.8)	
Not removed	1 (1.4)	1 (0.8)		1 (1.6)	1 (1.6)	
Total lymph nodes resected (*x* ± *s* ^a^)	19.84±8.38	20.02±7.47	0.881	19.77±8.53	19.66±7.63	0.939
Bronchial sleeve resection/bronchoplasty						
No	39 (56.5)	72 (59.5)	0.688	36 (56.3)	40 (62.5)	0.472
Yes	30 (43.5)	49 (40.5)		28 (43.8)	24 (37.5)	
Vascular sleeve resection/angioplasty						
No	58 (84.1)	113 (93.4)	0.039	54 (84.4)	59 (92.2)	0.169
Yes	11 (15.9)	8 (6.6)		10 (15.6)	5 (7.8)	
Pericardial excision						
No	62 (89.9)	118 (97.5)	0.038	58 (90.6)	63 (98.4)	0.052
Yes	7 (10.1)	3 (2.5)		6 (9.4)	1 (1.6)	
Surgical radicality						
Radical	63 (91.3)	110 (90.9)	0.964	58 (90.6)	60 (93.8)	0.605
Palliative/uncertain	5 (7.2)	10 (8.3)		5 (7.8)	3 (4.7)	
Exploration	1 (1.4)	1 (0.8)		1 (1.6)	1 (1.6)	
Radiographic tumor response						
CR/PR	54 (78.3)	88 (72.7)	0.399	49 (76.6)	46 (71.9)	0.544
SD/PD	15 (21.7)	33 (27.3)		15 (23.4)	18 (28.1)	
Pathological response						
MPR(CR)	34 (49.3)	23 (19.0)	<0.001	31 (48.4)	11 (17.2)	0.001
PR	24 (34.8)	71 (58.7)		22 (34.4)	38 (59.4)	
SD(PD)	11 (15.9)	27 (22.3)		11 (17.2)	15 (23.4)	
Adjuvant systemic therapy						
No	2 (2.9)	26 (21.5)	<0.001	2 (3.1)	14 (21.9)	<0.001
Chemo	9 (13.0)	82 (67.8)		9 (15.1)	42 (65.6)	
PD‐1 + Chemo	58 (84.1)	13 (10.7)		53 (82.8)	8 (12.5)	
First failure site						
Local	4 (28.6)	9 (19.6)	0.692	4 (28.6)	5 (20.0)	0.775
Distant	9 (64.3)	31 (67.4)		9 (64.3)	17 (68.0)	
Local + distant	1 (7.1)	6 (13.0)		1 (7.1)	3 (12.0)	

Abbreviations: Chemo, chemotherapy; ICU, intensive care unit; NSCLC, non‐small cell lung cancer; PD‐1 + Chemo, PD‐1 inhibitor plus chemotherapy; TRAEs, treatment related adverse events; VATS, video‐assisted thoracic surgery.

^a^ Variables were described by mean (*x*) and standard deviation (*s*).

Surgery was delayed beyond 42 days after the final neoadjuvant therapy in 10 patients (14.5%) from the PD‐1 + Chemo group because of TRAEs (6 patients), pulmonary infection (2 patients), and economic reasons or hesitation to undergo surgery (2 patients). Fourteen patients (11.6%) received surgery delayed beyond 42 days in the Chemo group because of AEs (8 patients), economic reasons or hesitation to undergo surgery (5 patients), and deep venous thrombosis (1 patient). No significant difference was detected in the interval between final neoadjuvant treatment and surgery between the two groups (*p* = 0.560). After PSM adjustment, the interval between final neoadjuvant treatment and surgery was still comparable between the two groups.

Radical resection (R0) was achieved in 63 (91.3%) and 110 (90.9%) patients, respectively, in the two groups (*p* = 0.964). There were five palliative or uncertain resections in the PD‐1 + Chemo group, including microscopically positive margin in two patients and positive highest lymph node in three. Palliative or uncertain resection was also performed in 10 patients from the Chemo group, including microscopically positive margin in three patients and positive highest lymph node in seven. Moreover, one patient in each group underwent explorative surgery, and both were abandoned because of advanced disease.

The percentages of VATS were 39.1% and 31.4% in the PD‐1 + Chemo and Chemo groups, respectively, while 29.0% and 23.1% of the patients in each group, respectively, received conversion thoracotomy (*p* = 0.187). Lobectomy was performed in 73.9% and 66.1% of patients, and bilobectomy in 14.5% and 24.0% for the PD‐1 + Chemo versus Chemo groups, respectively (*p* = 0.471). The two groups were also comparable for bronchial sleeve resection/bronchoplasty rate (43.5% vs. 40.5%, respectively, *p* = 0.688). However, vascular sleeve resection/angioplasty was performed in 15.9% of patients in the PD‐1 + Chemo group, which was more than in the Chemo group (6.6%; *p* = 0.039). Seven patients (10.1%) in the PD‐1 + Chemo group received pericardial resection, while only three (2.5%) in the Chemo group received pericardial resection (*p* = 0.038). No significant difference was found between the two groups in the total number of resected lymph nodes (mean: 19.84 ± 8.38 vs. 20.02 ± 7.47, *p* = 0.881).

However, after PSM, the statistical difference between the groups in vascular sleeve resection/angioplasty disappeared (*p* = 0.169), but the pericardial resection rate in the PD‐1 + Chemo group was still relatively higher (9.4% vs. 1.6%, *p* = 0.052), suggesting there may be a tendency for more complicated pulmonary resections following treatment with neoadjuvant PD‐1 inhibitors plus chemotherapy.

### 3.3 Pathological response

Radiographic response evaluation was performed in all patients before surgery, and the results showed CR/PR in 54 (78.3%) and 88 (72.7%) patients in the two groups, respectively (*p* = 0.399). After surgery, the final pathological analysis showed MPR in 34 patients (49.3%) in the PD‐1 + Chemo group, among whom 23 (33.3%) showed pCR, which was significantly higher than the MPR rate of 19.0% (23 patients) and pCR rate of 9.9% (12 patients) in the Chemo group (Table 2, *p* < 0.001). After further PSM analysis, the MPR rate in the PD‐1 + Chemo group was 48.4%, which remained significantly higher than that of the Chemo group (17.2%; *p* = 0.001). In subgroup analysis of MPR in the PD‐1 + Chemo group (Table 3), only histology was related with the MPR rate, with rates of 59.6% and 17.6% in squamous cell carcinoma and non‐squamous cell carcinoma, respectively (*p* = 0.003).

**TABLE 3:** Subgroup analysis of major pathological response (MPR) in population received neoadjuvant chemoimmunotherapy

Characteristics	MPR *N* = 34	Non‐MPR *N* = 35	*p*
Age, %			
<60	19 (51.4)	18 (48.6)	0.711
≥60	15 (46.9)	17 (53.1)	
Gender, %			
Male	33 (50.0)	33 (50.0)	0.575
Female	1 (33.3)	2 (66.7)	
Smoking habits, %			
Non‐smoker	3 (42.9)	4 (57.1)	0.722
Present/ex‐smoker	31 (50.0)	31 (50.0)	
Weight loss before treatment, %			
Yes	7 (46.7)	8 (53.3)	0.819
No	27 (50.0)	27 (50.0)	
Histology, %			
Squamous cell carcinoma	31 (59.6)	21 (40.4)	0.003
Non squamous cell carcinoma	3 (17.6)	14 (82.4)	
Tumour length, %			
<5 cm	15 (45.5)	18 (54.5)	0.543
≥5 cm	19 (52.8)	17 (47.2)	
cT stage, %			
T1–2	14 (50.0)	14 (50.0)	0.921
T3–4	20 (48.8)	21 (51.2)	
cN stage, %			
N0	3 (30.0)	7 (70.0)	0.142
N1	17 (63.0)	10 (37.0)	
N2	14 (43.8)	18 (56.3)	
cTNM stage, %			
IB–II	9 (52.9)	8 (47.1)	0.922
IIIA	16 (47.1)	18 (52.9)	
IIIB	9 (50.0)	9 (50.0)	
Neoadjuvant therapy cycles, %			
<3	28 (50.9)	27 (49.1)	0.591
≥3	6 (42.9)	8 (57.1)	
Platinum type, %			
Cisplatinum	4 (44.4)	5 (55.6)	0.756
Carboplatin	30 (50.0)	30 (50.0)	

Abbreviations: Chemo, chemotherapy; cN, clinical N stage before treatment; cT, clinical T stage before treatment; cTNM, clinical TNM stage before treatment; NSCLC, non‐small cell lung cancer; PD‐1 + Chemo, PD‐1 inhibitor plus chemotherapy.

### 3.4 Survival outcomes and prognostic factors

As of February 28, 2022, the median follow‐up of the PD‐1 + Chemo group was 18.6 months (range: 11.0–34.2 months), while the median follow‐up time was 22.4 months (range: 0.3–35.8 months) in the Chemo group. At the end of follow‐up, tumor recurrence occurred in 14 (20.3%) and 46 (38.0%) patients from the two groups, respectively (*p* = 0.011). In the PD‐1 + Chemo group, the first failure sites in the 14 patients included distant metastasis in nine (64.3%), local recurrence in four (28.6%), and concurrent local + distant recurrence in one (7.1%). In the Chemo group, the principal failure pattern was distant metastasis (31 patients, 67.4%), local recurrence (9 patients, 19.6%), and concurrent local + distant recurrence (6 patients, 13.0%; *p* = 0.692).

**FIGURE 1:**
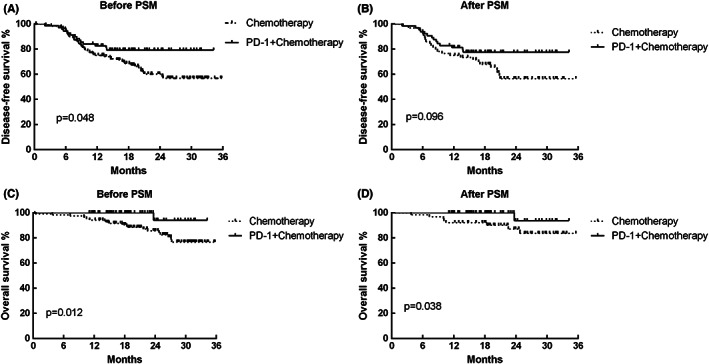
(A) The disease‐free survival (DFS) curves for programmed cell death protein‐1 (PD‐1) + Chemo group and Chemo group before propensity score matching analysis (PSM) (*p* = 0.048). (B) The DFS curves for PD‐1 + Chemo group and Chemo group after PSM (*p* = 0.096). (C) The overall survival (OS) curves for PD‐1 + Chemo group and Chemo group before PSM (*p* = 0.012). (D) The OS curves for PD‐1 + Chemo group and Chemo group after PSM (*p* = 0.038).

**FIGURE 2:**
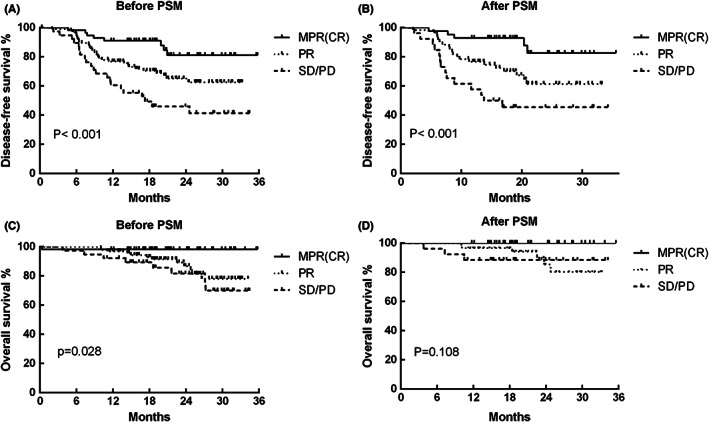
(A) The disease‐free survival (DFS) curves for different pathological response before propensity score matching analysis (PSM) (*p* < 0.001). CR, complete response; MPR, major pathological response; PD, progressive disease; PR, partial response; SD, stable disease. (B) The DFS curves for different pathological response after PSM (*p* < 0.001). (C) The overall survival (OS) curves for different pathological response before PSM (*p* = 0.028). (D) The OS curves for different pathological response after PSM (*p* = 0.108).

Overall, the 2‐year DFS rate was 79.3% in the PD‐1 + Chemo group, which was higher than that of Chemo group (60.2%; *p* = 0.048; Figure 1A). After PSM, the statistical significance disappeared (Figure 1B, *p* = 0.096); however, the survival curves were separate, indicating that neoadjuvant PD‐1 + chemotherapy might achieve longer tumor control. Other factors including histology, radical surgery, radiographic tumor response, pathological response, ypT stage, ypN stage, and ypTNM stage were also found to significantly correlate with DFS in univariate Cox analysis (Table 4). However, in multivariate analysis, which included significant factors identified by univariate analysis, only surgical radicality (hazard ratio [HR]: 2.954, 95% confidence interval (CI): 1.527–5.714, *p* = 0.001) and pathological response (MPR(CR) vs. SD(PD), HR: 0.248, 95% CI: 0.107–0.572, *p* = 0.001, Figure 2A,B) but not neoadjuvant therapeutic modality were found to be independent prognostic factors for DFS (Table 4).

**TABLE 4:** Univariate and multivariate analysis of DFS for 190 NSCLC patients treated with surgery following neoadjuvant PD‐1 + chemotherapy or chemotherapy alone

Characteristics	Univariate		Multivariate	
	HR (95% CI)	*p*	HR (95% CI)	*p*
Age (year): <60 versus ≥60	1.166 (0.702–1.937)	0.553		
Gender: male versus female	1.365 (0.587–3.175)	0.470		
Tumour location: central versus peripheral	1.690 (0.971–2.941)	0.064^a^		
Histology: SCC versus non‐SCC	1.734 (1.019–2.951)	0.043^a^		
cT stage: T1–2 versus T3–4	1.223 (0.737–2.032)	0.436		
cN stage: N0 versus N+	0.697 (0.353–1.376)	0.298		
cTNM stage: IB–II versus IIIA–B	1.969 (0.969–4.000)	0.061^a^		
Surgical radicality: palliative/exploration versus radical	4.033 (2.127–7.648)	<0.001^a^	2.954 (1.527–5.714)	0.001
Radiographic tumor response: CR/PR versus SD/PD	2.039 (1.211–3.433)	0.007^a^		
Pathological response: MPR(CR)	0.201 (0.089–0.451)	<0.001^a^	0.248 (0.107–0.572)	0.001
PR	0.520 (0.298–0.904)	0.021^a^	0.594 (0.337–1.046)	0.071
SD(PD)	Reference		Reference	
ypT stage: ypT0–2 versus ypT3–4	2.462 (1.352–4.483)	0.003^a^		
ypN stage: ypN0 versus ypN+	1.784 (1.073–2.964)	0.026^a^		
ypTNM stage: 0–II versus IIIA–B	1.204 (1.085–1.335)	<0.001^a^		
Neoadjuvant therapeutic modality: chemo versus PD‐1 + Chemo	0.818 (0.670–0.998)	0.048^a^		
Adjuvant systemic therapy: no	Reference			
Chemo	0.828 (0.400–1.712)	0.986		
PD‐1 + Chemo	0.993 (0.471–2.093)	0.834		

Abbreviations: Chemo, chemotherapy; CI, confidence interval; cN, clinical N stage before treatment; cT, clinical T stage before treatment; cTNM, clinical TNM stage before treatment; HR, hazard ratio; NSCLC, non‐small cell lung cancer; PD‐1 + Chemo, PD‐1 inhibitor plus chemotherapy; SCC, squamous cell carcinoma.

^a^ Factors included into multivariate analysis.

Furthermore, in the unweighted population, the 2‐year OS rate in the PD‐1 + Chemo group was 94.1%, which was significantly longer than that of the Chemo group (85.7%; *p* = 0.012; Figure 1C). The weighted population yielded 93.8% versus 87.1%, respectively (Figure 1D, *p* = 0.038). The 2‐year OS in patients who achieved MPR(CR) was 98.2%, which was significantly higher than that of PR (86.7%) and SD/PD (81.7%; *p* = 0.028; Figure 2C). After PSM, patients who achieved MPR still had the best survival outcomes, although the statistical significance disappeared (*p* = 0.108; Figure 2D).

### 3.5 Perioperative morbidity

TRAEs related to drugs before surgery were observed in 41 (59.4%) and 69 (57.0%) patients in the two groups, respectively (*p* = 0.748, Table 5). Myelosuppression, immunologic hepatitis/hepatic dysfunction, and vomiting/nausea were the most frequently observed AEs in both groups (Table 5). Hepatic dysfunction might be induced by PD‐1 inhibitors or chemotherapeutic agents, and it is difficult to differentiate in clinical practice. Thus, the frequencies of immunologic hepatitis and hepatic dysfunction were summarized into one group, with a relatively higher incidence in the PD‐1 + Chemo group (43.1% vs. 20.3%). Furthermore, five patients (7.2%) in the PD‐1 + Chemo group suffered from other immunologic AEs except immunologic hepatitis.

**TABLE 5:** Treatment related adverse events before surgery in both PD‐1 + Chemo and Chemo groups

Adverse events	PD‐1 + Chemo (*n* = 41, %)	Chemo (*n* = 69, %)
Myelosuppression	15 (36.6)	37 (53.6)
Immunologic hepatitis/hepatic dysfunction	14 (34.1)	14 (20.3)
Vomiting/nausea	4 (9.6)	13 (18.8)
Renal dysfunction	2 (4.9)	2 (2.9)
Numbness of extremities	1 (2.4)	2 (2.9)
Hypothyroidism	1 (2.4)	0
Immunologic pneumonitis	1 (2.4)	0
Immunologic myocarditis	1 (2.4)	0
Immunologic myositis	1 (2.4)	0
Skin rash	1 (2.4)	1 (1.4)

Abbreviations: Chemo, chemotherapy; PD‐1 + Chemo, PD‐1 inhibitor plus chemotherapy.

During surgery, there were three (4.3%) and five (4.1%) patients, respectively, in the two groups, who suffered from accidental intraoperative bleeding and received blood transfusions.

Postoperative complications within 30 days occurred in 15 (21.7%) and 42 (34.5%) patients in the two groups (*p* = 0.061), respectively, which are summarized in detail in Table 6. The principal complications in both groups included postoperative pneumonitis, prolonged air leak, and arrhythmia/heart failure, which were unrelated to the neoadjuvant therapeutic modality. Only one patient had immunological hepatitis after surgery in the PD‐1 + Chemo group. And one patient died 9 days after surgery because of bronchial anastomotic leakage and hemoptysis in the Chemo group, while no 90‐day mortality occurred in the PD‐1 + Chemo group. As a result, there was no statistically significant difference on postoperative morbidity and mortality in the two groups.

**TABLE 6:** Postoperative complications within 30 days in both PD‐1 + Chemo and Chemo groups

Complications	PD‐1 + Chemo (*n* = 15, %)	Chemo (*n* = 42, %)
Pneumonitis	4 (26.7)	16 (38.1)
Prolonged air leak	4 (26.7)	9 (21.4)
Haemothorax	0	2 (4.8)
Chyle	1 (6.7)	2 (4.8)
Bronchial anastomotic leakage	0	1 (2.4)
Pneumonitis and heart failure	1 (6.7)	0
Immunologic hepatitis/hepatic dysfunction	1 (6.7)	1 (2.4)
Arrhythmia/heart failure	2 (13.3)	5 (11.9)
Urinary retention	1 (6.7)	3 (7.1)
Vomiting/nausea	1 (6.7)	3 (7.1)

Abbreviations: Chemo, chemotherapy; PD‐1 + Chemo, PD‐1 inhibitor plus chemotherapy.

## 4 DISCUSSION

The impact of neoadjuvant immunotherapy on surgery and related outcomes has been poorly reported in previous studies.^9^ Thus, there exists an urgent need to identify the benefit–risk profile, potential intraoperative technique challenges, and surgery‐related complications in large‐scale real‐world settings. In this retrospective study that enrolled a relatively large population, several encouraging results were identified in accordance with previous studies.^6^ The MPR rate in the PD‐1 + Chemo group was 49.3%, which was significantly higher than that of the Chemo group (19.0%), with pCR rates of 33.3% versus 9.9%, respectively. For the first time, our study has identified that intraoperative difficulties might be increased after neoadjuvant PD‐1 inhibitors plus chemotherapy because of the increased frequency of pericardial resection and other complicated pulmonary resections. Meanwhile, perioperative morbidities were still comparable between the two groups.

As one of the most commonly used surrogate endpoints for predicting survival,^14^ our study found a positive MPR result and met the primary endpoint. Our data from a real‐world setting confirmed that neoadjuvant chemoimmunotherapy yielded promising efficacy, with a 30.3% increased MPR rate and a 23.4% increased pCR rate compared with Chemo alone. After PSM, the MPR rate of the PD‐1 + Chemo group was 48.4%, which was significantly higher than that of the Chemo group (17.2%). In accordance with previous reports,^6^, ^7^, ^15^, ^16^, ^17^, ^18^, ^19^, ^20^, ^21^, ^22^, ^23^, ^24^ MPR rates ranged from 27% to 86% in the chemoimmunotherapy modality, while the range was 8.9% to 16% with neoadjuvant chemotherapy alone. Several studies^16^, ^22^ have reported relatively higher MPR rates in squamous cell carcinoma patients. In our subgroup analysis, the MPR rate was 59.6% in squamous cell carcinoma, which was also significantly higher than in the non‐squamous cell carcinoma group (17.6%), while no correlation was identified between other factors and the MPR rate, including age, gender, smoking habits, weight loss before treatment, tumor length, cT stage, cN stage, cTNM stage, neoadjuvant therapy cycles, and platinum type.

One of the controversies surrounding the use of neoadjuvant therapy is whether potentially curative surgery will be delayed or even if patients will fail to undergo surgery because of the severe AEs related to drugs or disease progression before surgery. In previous large multicenter studies,^7^, ^16^, ^17^, ^25^, ^26^, ^27^ 1% to 7% of patients failed to receive surgery because of severe AEs, 0% to 8% because of progressive disease on radiographic evaluation after neoadjuvant chemoimmunotherapy, and 5% to 10% were identified as unresectable during surgery. In this study, there were 10 (14.5%) and 14 (11.6%) patients, respectively, who received surgery delayed beyond 42 days after the final neoadjuvant therapy in the PD‐1 + Chemo and Chemo groups, which is in accordance with previous results,^7^, ^27^ suggesting that neoadjuvant PD‐1 + Chemo did not delay surgery. Only one patient was identified as unresectable during surgery because of advanced disease in each group. However, over 90% of patients achieved radical resection in both groups in this study; similarly, 83% to 100% of patients underwent successful R0 resection after neoadjuvant chemoimmunotherapy in previous reports.^16^, ^17^, ^18^, ^20^ Unfortunately, the data on patients who failed to receive surgery were absent because this study only included patients who received surgery after neoadjuvant therapy.

Considering the possibility of fibrosis and adhesion after neoadjuvant therapy, it is still debatable whether the complexity of pulmonary resection was increased or not. In the NEOSTAR trial,^28^ surgeons believed that 40% of operations following neoadjuvant Nivolumab or Ipilimumab + nivolumab were more difficult than anatomical lobectomies for stage I lung cancer without previous treatment. However, the details of intraoperative difficulties have been poorly described in previous large‐scale studies. In anecdotal reports,^22^, ^23^, ^24^, ^29^, ^30^, ^31^ 16.1%–30% of patients received sleeve lobectomy after chemoimmunotherapy, and even one patient received autogenous lobar transplantation.^29^ Deng et al.^30^ reported that 48.4% of patients (15/31) had dense adhesions in the fissure or nodal stations after neoadjuvant chemoimmunotherapy. Liang et al.^29^ identified greater destruction of elastic fiber of the blood vessels and vascular wall degeneration following neoadjuvant chemoimmunotherapy compared with the state after chemotherapy. In this large‐scale retrospective analysis, more patients received vascular sleeve resection/angioplasty and pericardial resection in the PD‐1 + Chemo group, while the bronchial sleeve resection/bronchoplasty rate was comparable between the two groups. Although the statistical difference in the vascular sleeve resection/angioplasty rate between the groups disappeared after PSM, the pericardial resection rate in the PD‐1 + Chemo group was still relatively higher. To the best of our knowledge, this is the first report to show that more complicated pulmonary resections might be performed following neoadjuvant chemoimmunotherapy. However, no significant difference was identified in other intraoperative factors, including surgical approach, extent of pulmonary resection, and total lymph nodes resected, proving that the addition of PD‐1 inhibitors as neoadjuvant therapy did not significantly change the administration of pulmonary resection.

In this study, the 2‐year DFS rate in the PD‐1 + Chemo group was 79.3%, which was higher than that of the Chemo group (60.2%). In the NADIM study, which included 46 patients with stage IIIA NSCLC, the 2‐year PFS rate was 77.1%, and the OS rate was 89.9%.^18^ Zhai et al.^20^ reported in a retrospective analysis that the 24‐month PFS was 45.8% and the OS rate was 79.9% after neoadjuvant nivolumab and chemotherapy. In another real‐world analysis from China,^21^ the 2‐year DFS rate was 81.8% in the MPR groups, while it was 37.3% in the non‐MPR group; multivariate analysis identified maximal tumor length of the specimen, ypN1‐2, and non‐MPR pathological evaluation as independent factors affecting poor prognosis. Our analysis also confirmed that surgical radicality and pathological response were both independent prognostic factors for DFS. Although neoadjuvant treatment modality was not an independent prognostic factor in this study, the significantly higher MPR rate after neoadjuvant chemoimmunotherapy indicated that neoadjuvant PD‐1 + Chemo might have an indirect effect on patient prognosis. Regarding the 2‐year OS, patients who received PD‐1 + Chemo and achieved MPR(CR) also achieved better results. However, because the follow‐up time was too limited and the OS data were immature, further analysis of prognostic factors was not conducted in this study.

Perioperative morbidity is one of the principal concerns in surgical treatment following neoadjuvant chemoimmunotherapy. In previous studies,^20^, ^32^, ^33^ approximately 4.8% to 8.9% of patients suffered from major intraoperative bleeding, in accordance with that of 4.3% in this study. As reported in the NADIM study^18^ and other reports,^25^, ^31^, ^32^, ^33^ the most frequent complications within 30 days after surgery were pneumonitis, prolonged air leakage, and cardiac arrhythmia, while the incidence of immunologic AEs was relatively low. In this study, only one patient died within 9 days after surgery in the Chemo group, while no 90‐day mortality occurred in the PD‐1 + Chemo group. Also, no statistically significant difference on postoperative morbidity and mortality was detected between the two groups, proving that neoadjuvant PD‐1 inhibitors plus chemotherapy had no negative effect on postoperative recovery and did not increase perioperative morbidity.

However, attention should be paid to the limitations of this study: First, although PSM was used to balance heterogeneity between the groups, selection biases were inevitable because of the retrospective nature and limited sample size. Second, OS is the gold standard efficacy endpoint in cancer trials, but the follow‐up time was too short to make OS the primary endpoint of this study. It is necessary to make mature and stable conclusions after a longer follow‐up time in future studies. Third, because there were six different PD‐1 inhibitors used in this study, differences in the efficacy and adverse effects of different drugs^25^ might exist, and subgroup analysis should be performed. Finally, detailed information on predicting biomarkers including PD‐L1 expression was absent and its influence on DFS and OS requires further investigation. Therefore, more large‐scale randomized clinical trials and real‐world comparative studies should be conducted to clarify the advantages and disadvantages of neoadjuvant chemoimmunotherapy in early‐stage NSCLC.

In conclusion, this real‐world study confirmed that neoadjuvant chemoimmunotherapy is safe and feasible, with higher MPR and pCR rates, as well as favorable DFS compared with chemotherapy alone. Surgical complexity might be increased in certain patients, with comparable perioperative morbidity and mortality.

## AUTHOR CONTRIBUTIONS

Conception and design: B Zhang, H Xiao, Q Xiao, W Wang, X Pu, C Zhou. Administrative support: B Zhang, W Wang. Provision of study materials or patients: B Zhang, X Pu, D Yang, C Zhou, X Li, W Wang, Q Xiao. Collection and assembly of data: B Zhang, H Xiao, Q Xiao. Data analysis and interpretation: B Zhang, H Xiao, Q Xiao. Manuscript writing: All authors. Final approval of manuscript: All authors.

## FUNDING INFORMATION

This study was supported in part by the Natural Science Foundation of Hunan Province (2019JJ40179); Innovation Guide Program of Medical Technology in Hunan Province (2020SK51111); Scientific Research Project of Hunan Provincial Health and Family Planning Commission (20201743); Hunan Clinical Medical Research Center of Accurate Diagnosis and Treatment for Esophageal Carcinoma (2020SK4005); and Hunan Cancer Hospital Climb Plan (ZX2020005).

## CONFLICT OF INTEREST

The authors declare no potential conflicts of interest.

## Data Availability

The data that support the findings of this study are available from the corresponding author upon reasonable request.
